# Type 1 diabetic mellitus patients with increased atherosclerosis risk display decreased *CDKN2A/2B/2BAS* gene expression in leukocytes

**DOI:** 10.1186/s12967-019-1977-1

**Published:** 2019-07-12

**Authors:** Sergio Martínez-Hervás, Verónica Sánchez-García, Andrea Herrero-Cervera, Ángela Vinué, José Tomás Real, Juan F. Ascaso, Deborah Jane Burks, Herminia González-Navarro

**Affiliations:** 1 Endocrinology and Nutrition Department Hospital Clínico Universitario. Department of Medicine, University of Valencia, 46010 Valencia, Spain; 2 INCLIVA Institute of Health Research, Avda. Menéndez Pelayo, 4, 46010 Valencia, Spain; 3 CIBER Diabetes and Associated Metabolic Diseases (CIBERDEM), 28029 Madrid, Spain; 4 Príncipe Felipe Research Center (CIPF), 46012 Valencia, Spain; 5 Department of Didactics of Experimental and Social Sciences, University of Valencia, 46010 Valencia, Spain

**Keywords:** Type 1 diabetes, Inflammation, Cardiovascular risk, T cells

## Abstract

**Background:**

Type 1 diabetes mellitus (T1DM) patients display increased risk of cardiovascular disease (CVD) and are characterized by a diminished regulatory T (Treg) cell content or function. Previous studies have shown an association between decreased *CDKN2A/2B/2BAS* gene expression and enhanced CVD. In the present study the potential relationship between *CDKN2A/2B/2BAS* gene expression, immune cell dysfunction and increased cardiovascular risk in T1DM patients was explored.

**Methods:**

A cross-sectional study was performed in 90 subjects divided into controls and T1DM patients. Circulating leukocyte subpopulations analysis by flow cytometry, expression studies on peripheral blood mononuclear cell by qPCR and western blot and correlation studies were performed in both groups of subjects.

**Results:**

Analysis indicated that, consistent with the described T cell dysfunction, T1DM subjects showed decreased circulating CD4+CD25+CD127− Treg cells. In addition, T1DM subjects had lower mRNA levels of the transcription factors FOXP3 and RORC and lower levels of IL2 and IL6 which are involved in Treg and Th17 cell differentiation, respectively. T1DM patients also exhibited decreased mRNA levels of *CDKN2A (variant 1 p16*^*Ink4a*^), *CDKN2A (p14*^*Arf,*^ *variant 4*), *CDKN2B* (*p15*^*Ink4b*^) and *CDKN2BAS* compared with controls. Notably, T1DM patients had augmented pro-atherogenic CD14++CD16+-monocytes, which predict cardiovascular acute events and enhanced common carotid intima-media thickness (CC-IMT).

**Conclusions:**

Decreased expression of *CDKN2A/2B/2BAS* in leukocytes associates with increased CC-IMT atherosclerosis surrogate marker and proatherogenic CD14++CD16+ monocytes in T1DM patients. These results suggest a potential role of *CDKN2A/2B/2BAS* genes in CVD risk in T1DM.

**Electronic supplementary material:**

The online version of this article (10.1186/s12967-019-1977-1) contains supplementary material, which is available to authorized users.

## Background

Type 1 diabetes mellitus is an autoimmune disease characterized by a deficiency of insulin production by the pancreas. Increased susceptibility to T1DM is determined by a combination of several genetic and environmental factors. Thus, variants of human leukocyte antigen (HLA) genes which influences antigen presentation during thymic cell selection processes and peripheral activation of the immune response have been associated with T1DM pathogenesis [1]. The disease results from the destruction of insulin-producing β cells by autoreactive effector CD4+ and CD8+ T cells, which initially infiltrate the islets producing insulitis, in response to pancreatic islet autoantigens [2]. These autoreactive T cell responses to autoantigens are present before the clinical onset of the disease. In healthy conditions T cell responses are balanced by regulatory mechanisms such as immunological tolerance, but have a reduced functional capacity during disease development [1]. One key player in immunological tolerance is a CD4+ T cell sub-population, regulatory T (Treg) cells, whose defective function has been suggested to contribute to T1DM development. It has been shown that T1DM individuals display apoptosis-prone Treg cells in the blood. Furthermore, analysis of lymphocytic islet infiltration from T1DM patient donors revealed autoreactive T cells with an effector phenotype, such as CD4+ T helper (Th) 1, an abnormal expansion of proinflammatory effector Th17 cells and diminished Treg cell content or function [3].

Despite glycemic control through restoration of insulin levels, T1DM subjects display increased cardiovascular disease (CVD) similar to that observed in type 2 diabetes mellitus (T2DM) subjects [4, 5]. However, unlike T2DM there is a lack of understanding of the risk in T1DM patients in current intensive glycemic control management guidelines. Many T1DM patients might display metabolic abnormalities that promote characteristics associated with enhanced CVD risk such as a chronic proinflammatory state. On the other hand, as mentioned above, T1DM subjects exhibit deranged T cell function which is also associated with increased CVD risk.

In fact, atherosclerosis progression, the main cause of CVD, is facilitated by an unbalanced interplay of different CD4+ T cell subsets, which promote plaque lesion formation in the vascular bed. Proinflammatory effector CD4+ Th1 and Th17 subsets are proatherogenic while Treg cells, which are a minor population in plaques, suppress Th cell activity in lesions. Moreover, Treg cells promote an anti-inflammatory and pro-resolving macrophage phenotype within atheromas [6]. Thus, altered Treg/Th17 ratio in circulating leukocytes favor atherosclerosis progression and human patients with vulnerable atheroma plaques and coronary artery disease (CAD) display reduced circulating Treg cells [7–9].

We have recently shown that CAD complications in T2DM subjects associated with changes in immune cell homeostasis and with diminished expression of *CDKN2A/2B/2BAS* genes [10]. These genes encode for the tumour suppressors p16^Ink4a^, ARF and p15^Ink4b^ and the regulatory antisense non-coding RNA named ANRIL (antisense noncoding RNA in the INK4 locus) [11]. Previous genome-wide studies linked single nucleotide polymorphisms (SNPs) in *CDKN2A/2B/2BAS* genes to increased risk of T2DM and CVD [11]. Functional studies also indicated that upregulation of these genes might be a therapeutic tool to modulate insulin resistance and atherosclerosis [12–17].

In light of the above research, in the present study we investigated whether deranged immune systems in T1DM individuals could be related to atherosclerosis surrogate markers and altered expression of *CDKN2A/2B/2BAS* genes.

## Materials and methods

### Subjects

The study was performed in accordance with the ethical principles of the Helsinki Declaration, and was approved by the Hospital Clínico Universitario de Valencia ethics committee. All subjects gave written informed consent. For the study 90 unrelated individuals attending the outpatient clinic and selected over 18 months by the opportunistic sampling method, were divided into controls and T1DM subjects. T1DM was diagnosed when fasting plasma glucose was ≥ 126 mg/dL or when HbA1C was ≥ 6.5% or when a patient had classic symptoms of hyperglycemia or hyperglycemic crisis, with a random plasma glucose ≥ 200 mg/dl, in accordance with the ADA criteria for T1DM [18] together with positivity of autoantibodies.

### Human carotid artery ultrasound evaluation for common carotid intima-media thickness (CC-IMT)

A standardized imaging protocol was used for common carotid intima-media thickness (CC-IMT) measurements in agreement with the Mannheim consensus [19]. B-mode ultrasound imaging of the right and left carotid arteries was performed using a Siemens Sonoline G40 instrument equipped with 7 to 10-MHz broadband linear array transducers as previously described [20]. With the carotid dilatation and flow dividers as anatomic landmarks, the sonographer obtained high-resolution images of the common carotid (1 cm proximal to the bifurcation), the bifurcation (between dilatation and flow divider), and the internal carotid (1 cm distal to the flow divider). Carotid arteries and bifurcation were examined for presence of atherosclerotic plaques defined as described [20]. An experienced sonographer (S.M.-H.) performed all examinations. Intraobserver variability was examined in 20 subjects. The coefficient of variability of mean CC-IMT was 5.2%.

### Human plasma parameters analysis and enzyme-linked immunosorbent assay (ELISA)

Samples were collected after 12–14 h fasting from an antecubital vein in tubes containing EDTA (BD Vacutainer) and centrifuged within 4 h. Plasma was stored at 4 °C and biochemical parameters were measured by standard clinical methodology. Insulin was determined by radioimmunoassay. For ELISA heparinized human whole blood (10 U heparin/ml) was obtained, plasma was isolated and cytokines were measured using the human MCP1, TNFα, TGFβ, IL2, IL6 and IL17 DuoSet ELISA (R&D Systems, UK).

### Gene expression analysis by quantitative real-time PCR (qPCR)

Human peripheral blood mononuclear cells (PBMCs) were isolated from whole blood of patients with Lymphoprep (Axis Shield PoC, Norway) to obtain RNA using TRIzol (Invitrogen). RNA (0.5-1 μg) was retrotranscribed with cDNA Synthesis kit and amplified with Luminars Color-qPCR Master MIX (Fermentas) using a thermal Cycler 7900Fast System (Applied Biosystems). The primers were (Forward: Fw; Reverse: Rv): human *CDKN2A* (*splice variant 4*, *p14*^*Arf*^): Fw 5′CCCTCGTGCTGATGCTACTG3′ and Rv 5′CATCATGACCTGGTCTTCTAGGAA3′; human *CDKN2A* (*splice variant 1*, *p16*^*Ink4a*^): Fw 5′GGGGGCACCAGAGGCAGT3′ and Rv 5′ GGTTGTGGCGGGGGCAGTT3′; human *CDKN2B (p15*^*Ink4b*^*):* Fw 5′GGCAGTCGATGCGTTCACT3′ and Rv 5′AGGGCCTAAGTTGTGGGTTCA3′; human *CDKN2BAS:* Fw 5′CATGGTGGCAGCAAGAGAAAA3′ and Rv 5′TGATGGGTTTATCAGAGGTTTCC3′; human *GAPDH*: Fw 5′ACCACAGTCCATGCCATCAC3′ and Rv 5′TCCACCACCCTGTTGCTGTA3′; human *FOXP3*: Fw 5′GTGGCCCGGATGTGAGAAG3′ and Rv 5′GGAGCCCTTGTCGGATGATG3′; human *GATA3*: Fw 5′TTAGAGCCCTGCTCGATGCT3′ and Rv 5′CATGATACTGCTCCTGCAAAAATG3′; human *TBET*: Fw 5′TGCTCCAGTCCCTCCATAAGTAC3′ and Rv 5′TCTGGCTCTCCGTCGTTCAC3′; human *RORC*: Fw 5′GAAGTGGTGCTGGTTAGGATGTG3′ and Rv 5′CCACCGTATTTGCCTTCAAAA3′; human *SOCS1*: Fw 5′CCCTGGTTGTTGTAGCAGCTT3′ and Rv 5′GGTTTGTGCAAAGATACTGGGTATATG3′; human *SOCS3*: Fw 5′TGGGACGATAGCAACCACAA3′ and Rv 5′CGAAGTGTCCCCTGTTTGGA3′. Results were analyzed with the provided software. mRNA levels were normalized with the endogenous control and with the relativized to control group mRNA levels.

### Western blot analysis

Human PBMC pellets were homogenized in the presence of ice-cold lysis TNG buffer (Tris–HCl 50 mM, pH7.5, NaCl 200 mM, Tween-20 1%, NP-40 0.2%) supplemented with protease inhibitor Complete Mini cocktail, PhosSTOP Phosphatase Inhibitor Cocktail (Roche, Germany), 2 Mm PhenylMethylSulfonylFluorid (PMSF), ß-glycerol phosphate 50 mM and 200 µM Na3VO (Sigma). Protein extracts (35-50 µg) were prepared with Laemmli buffer (5 min, 95 °C) and subjected to 12% w/v polyacrylamide gel electrophoresis and western blot analysis as described [10]. The following primary and secondary antibodies were used: anti-CDK4 (sc-260, SantaCruz), anti-p21 (sc-397 SantaCruz), anti-p27 (610242, BD), anti-β-Actin (A5441, Sigma), anti-mouse IgG-HRP (sc-2005, SantaCruz) and anti-rabbit IgG-HRP (sc-2004, SantaCruz). The immunocomplexes were detected with ECL Plus (ThermoFisher).

### Flow cytometry measurements

To characterize leukocytes 10 µl of heparinized whole blood from human samples cells were incubated (30 min, RT) with anti-CD3-APC, anti-CD69-PE, anti-CD14-V450, anti-CD16-PerCP-Cy5.5 and Regulatory T cell Cocktail (all from BD Pharmigen). All stainings were followed by incubation with Facs Lysing solution (BD) for 15-20 min before flow cytometry analysis using FACSVerse Flow cytometers (BD Biosciences, USA).

### Statistical analysis

Quantitative variables appear as mean ± sem and correlations as individual points. Differences were assessed by Student´s t-test or Mann–Whitney U test and correlation studies by the Spearman correlation coefficient (GraphPad Prism 5.03, CA, USA). Statistical significance was set at p ≤ 0.05.

## Results

### Characterization of human subjects with T1DM and controls

Patients’ demographic and clinical characteristics are summarized in Table 1. Gender and age distribution were similar between T1DM and control groups. Compared with controls, T1DM had higher levels of systolic blood pressure (p < 0.05), BMI index (p < 0.01), fasting glucose (p < 0.001), HbA1c (p < 0.001) and lower levels of HDL-C (p < 0.05). No differences between the two groups were observed in total-cholesterol (CT), LDL-cholesterol, apoB or in triglycerides. Compared with controls, T1DM also displayed augmented CC-IMT, the surrogate atherosclerotic marker, (p < 0.03) indicating increased CVD risk. T1DM subjects had a higher prevalence (19.35% vs 0%) of statin treatment, as lipid-lowering therapy, than controls.

**Table 1:** Demographic and clinical characteristics of human subjects

	Controls (n = 48)	T1DM (n = 29)
Age (years)	32.3 ± 1.4	32.5 ± 1.7
SBP (mmHg)	116.2 ± 1.8	136.0 ± 1.8*
DBP (mmHg)	73.2 ± 1.1	74.2 ± 1.3
Glucose (mg/dL)	87.7 ± 1.6	160.3 ± 13.6***
HbA1c (%)	5.3 ± 0.1	7.6 ± 0.2***
BMI	23.3 ± 0.6	25.5 ± 0.5
Total cholesterol (mg/dL)	182.2 ± 4.1	175.7 ± 5.4
HDL-C (mg/dL)	61.46 ± 1.7	56.8 ± 1.5
LDL-C (mg/dL)	116.12 ± 3.2	114.3 ± 4.5
apoB (mg/dL)	84.88 ± 2.7	84.77 ± 3.4
Triglycerides (mg/dL)	75.9 ± 4.3	67.7 ± 4.7
CC-IMT (mm)	0.41 ± 0.01	0.46 ± 0.01**
% of lipid-lowering therapies (statins)	0	19.35***

*SBP* systolic blood pressure, *DBP* diastolic blood pressure, *BMI* body mass index, *LDL-C* low density lipoprotein cholesterol, *HDL-C* high density lipoprotein cholesterol, *apoB* apolipoprotein B, *CC-IMT* common carotid intima-media thickness

* p < 0.05; ** p < 0.01; *** p < 0.001. Statistical significance was assessed by Mann–Whitney U test

As expected CC-IMT positively correlated with age (p < 0.03), BMI (p < 0.04), HbAc1 (p < 0.02), glucose (p < 0.01), LDL-C (p < 0.03) and apoB levels (p < 0.02) (Additional file 1: Table S1).

### Circulating leukocyte characterization in T1DM and control human subjects

**Fig. 1:**
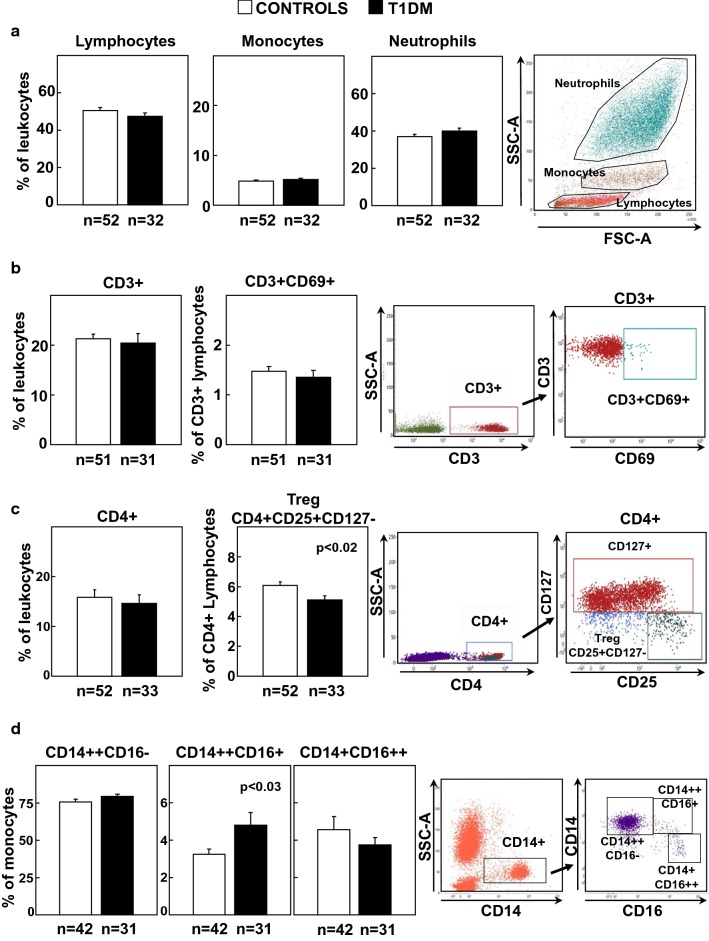
Characterization of circulating leukocytes in control and T1DM individuals. **a** Analysis of circulating lymphocytes, monocytes and neutrophils in both groups of subjects. **b** Circulating levels of CD3+ and CD3+CD69+ T-lymphocytes. **c** Circulating percentages of CD4 + T and CD4+ CD25+CD127−Treg cells. **d** Percentages of CD14 ++CD16-, CD14++CD16+ and CD14+ CD16++ monocytes relative to total monocytes. Statistical analysis was performed using Student’s t-test

Leukocyte analysis revealed no differences in the percentage of total lymphocytes, monocytes or neutrophils (Fig. 1a). Likewise, no differences were observed in the levels of total CD3 + T or activated CD3+ CD69+ T-lymphocytes between controls and T1DM patients (Fig. 1b). However, the percentage of CD4+ CD25+ CD127− Treg cells was significantly reduced in T1DM compared with controls (Fig. 1c, p < 0.02) without changes in total CD4+ T cells. Analysis of circulating monocytes into classical CD14++, intermediate CD14++ CD16+ and non-classical CD14+CD16++ subsets [21, 22], demonstrated an increase in the proinflammatory CD14++CD16+ subpopulation in T1DM patients compared with controls (Fig. 1d, p < 0.03).

### *CDKN2A/2B/2BAS* gene expression levels are altered in human subjects exhibiting T1DM

**Fig. 2:**
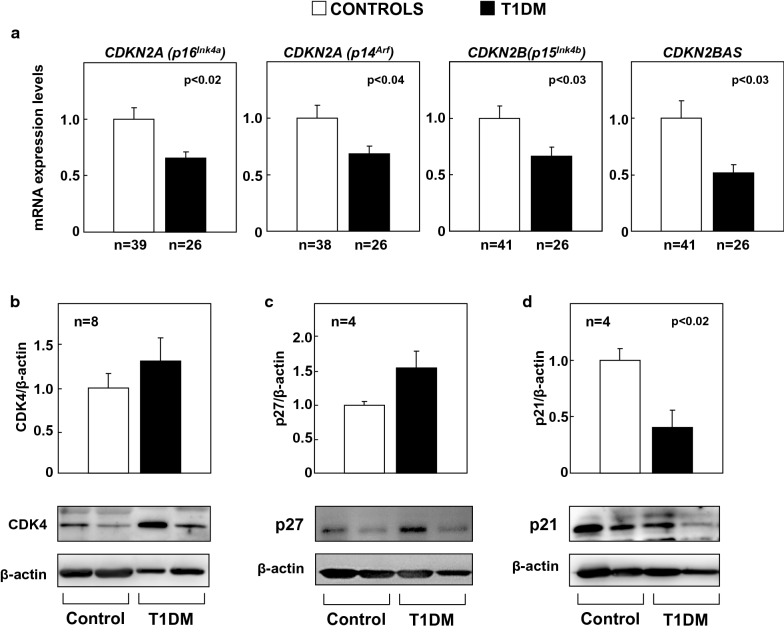
Expression analysis in PBMCs from control and T1DM human subjects. **a** mRNA expression levels of *CDKN2A(p16*^*Ink4a*^*)*, *CDKN2A(p14*^*Arf*^*)*, *CDKN2B* and *CDKN2BAS* normalized with the endogenous *GAPDH* mRNA levels and relativized to control group levels. Protein expression of (**b**) CDK4, (**c**) p27 and (**d**) p21 in PBMCs from human controls and T1DM patients. Protein levels were normalized to β-actin protein levels. Representative blots are shown below the quantifications. Statistical analysis was performed using Student’s t-test

Expression analysis in PBMCs showed lower mRNA levels of the *CDKN2A* (*splice variant 1, p16*^*Ink4a*^), of *CDKN2A* (*splice variant 4, p14*^*Arf*^), and *CDKN2B* (*p15*^*Ink4b*^), *CDKN2BAS* genes in T1DM individuals compared with controls (Fig. 2a, *CDKN2A (p16*^*Ink4a*^*)*, p < 0.02; *CDKN2A (p14*^*Arf*^) p < 0.04; *CDKN2B* (*p15*^*Ink4b*^) p < 0.03; *CDKN2BAS* p < 0.03). Analysis of other cell-cycle-related inhibitors showed decreased levels of p21 (p < 0.02) in T1DM individuals (Fig. 2d) compared with controls and no changes in CDK4 or p27 proteins (Fig. 2b, c).

Correlation studies of *CDKN2A/2B/2BAS* gene showed a negative correlation of *CDKN2A* (*p16*^*Ink4a*^) mRNA expression with HbA1C levels (Table 2, p < 0.05). Similarly, a negative correlation was found between *CDKN2B* (*p15*^*Ink4b*^) mRNA expression and glucose levels (Table 2, p < 0.03).

**Table 2:** Correlation between HbA1C and glucose levels and mRNA expression levels of the indicated genes

	HbA1C	
	Rho spearman	p value
*CDKN2A (p16* ^*Ink4a*^ *)*	− 0.2721	0.0445*
*CDKN2A (p14* ^*Arf*^ *)*	− 0.2045	0.1341
*CDKN2B (p15* ^*Ink4b*^ *)*	− 0.2324	0.074
*CDKN2BAS*	− 0.1738	0.1843

	Glucose	
	Rho spearman	p value
*CDKN2A (p16* ^*Ink4a*^ *)*	− 0.0929	0.4919
*CDKN2A (p14* ^*Arf*^ *)*	− 0.0526	0.6973
*CDKN2B (p15* ^*Ink4b*^ *)*	− 0.2802	0.0274*
*CDKN2BAS*	− 0.1214	0.3474

* p < 0.05; Statistical significance was assessed by non-parametric Spearman correlation coefficient

### Altered T cell subsets in PBMCs from human T1DM patients

T1DM is characterized by deranged balance of CD4+ Th cellular subsets, whose fate is determined by the expression of their corresponding transcription factors and activation of the different cytokine-induced JAK/STAT(signal transducer and activator of transcription)-signaling pathways. Expression of the above were therefore analyzed in T1DM subjects and controls.

**Fig. 3:**
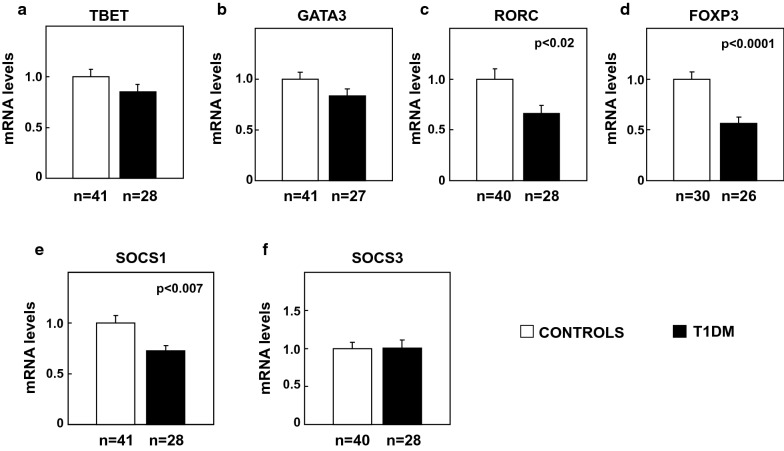
Analysis of the CD4 + T-cell differentiation signaling pathways in PBMCs from control and T1DM human subjects. mRNA expression levels of *TBET* (**a**), *GATA3* (**b**), *RORC* (**c**), *FOXP3* (**d**), *SOCS1* (**e**) and *SOCS3* (**f**). mRNA levels were normalized with the endogenous *GAPDH* mRNA levels and relativized to control group levels. Statistical analysis was performed using Student’s t-test

Expression analysis showed a marked reduction in *FOXP3* expression, a key transcription factor for Treg cell function, in T1DM subjects compared with controls (Fig. 3d, p < 0.0001). Similarly, analysis of the mRNA levels of *RORC*, the transcription factor expressed in Th17 cells was also diminished in T1DM subjects (Fig. 3c, p < 0.02). No changes were observed in *TBET* or *GATA3* (Fig. 3a, b). Given that JAK-dependent pathways can be directly inhibited by *SOCS1* and *SOCS3* proteins [23], these were also analyzed. T1DM patients showed a marked decrease in the expression of *SOCS1* mRNA (Fig. 3e, p < 0.007) and no changes in *SOCS3* mRNA levels (Fig. 3f) compared with controls.

Inverse correlations were found between mRNA levels of *FOXP3* and *SOCS*1 and HbA1c  % (Additional file 1: Table S2 p < 0.007 and p = 0.05 respectively) as well as with glucose levels (Additional file 1: Table S2, p < 0.05 and p < 0.004, respectively).

**Fig. 4:**
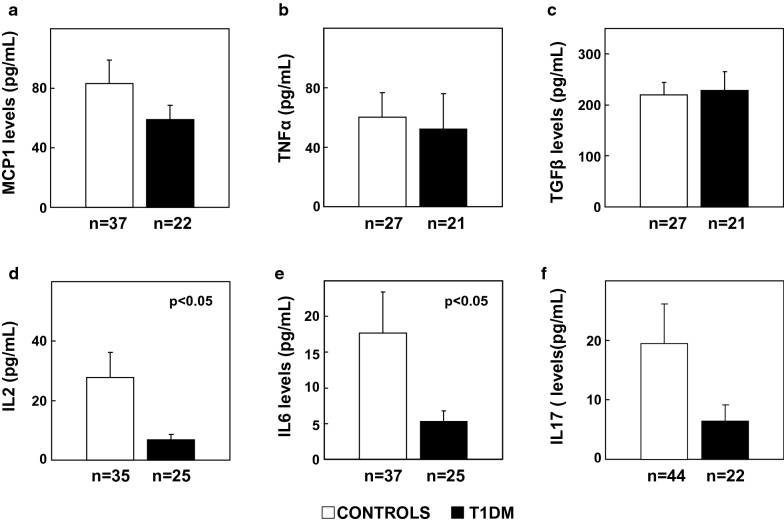
Circulating cytokine levels in control and T1DM human subjects. Plasmatic circulating levels of **a** MCP1, **b** TNFα, **c** TGFβ, **d** IL2, **e** IL6 and **f** IL17. Statistical analysis was performed using Student’s t-test

Circulating cytokine plasma analysis showed no changes in MCP1, TNFα or TGFβ levels between T1DM patients and controls (Fig. 4a, b). IL2 and IL6 were significantly decreased in T1DM compared with controls (Fig. 4d, e, p < 0.05 both) and no changes were observed in IL17 between controls and T1DM (Fig. 4f).

**Fig. 5:**
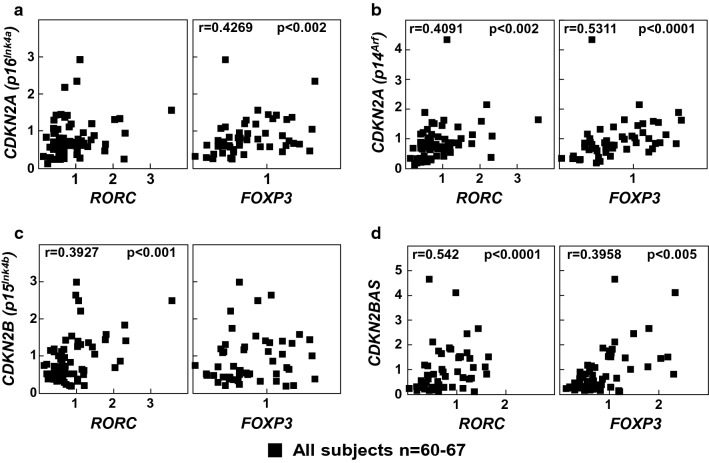
Correlation studies between of *CDKN2A/2B/2BAS* genes and transcription factors involved in T cell differentiation. Correlation of *CDKN2A(p16*^*Ink4a*^*)* **a**, *CDKN2A(p14*^*Arf*^*)* **b**, *CDKN2B* **c**, and *CDKN2BAS* **d** mRNA levels with *RORC* and *FOXP3* mRNA levels. Statistical analysis was performed using non-parametric Spearman correlation coefficient

**Fig. 6:**
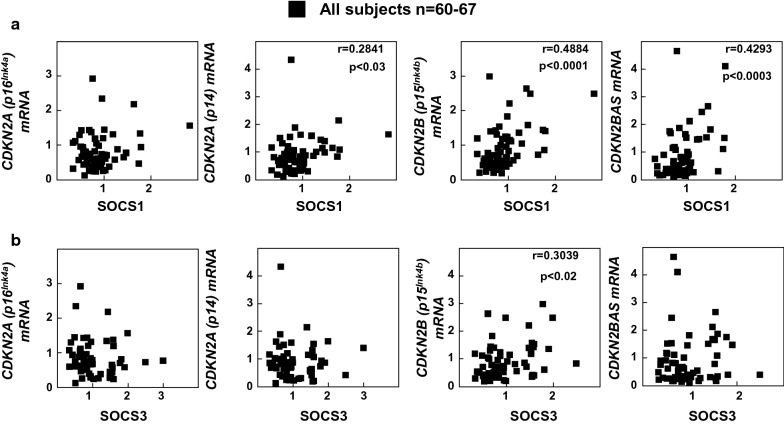
Correlation studies in all human subjects. Correlation of *CDKN2A/2B/2BAS* mRNA levels with **a** *SOCS1* and **b** *SOCS3* mRNA levels. Statistical analysis was performed using non-parametric Spearman correlation coefficient

Moreover, mRNA levels of *CDKN2A* (p16^Ink4a^) positively correlated with *FOXP3* levels (Fig. 5a, p < 0.002) while *CDKN2B* mRNA levels correlated with *RORC* (p < 0.001) mRNA levels (Fig. 5c). *CDKN2A* (p14^Arf^) and *CDKN2BAS* mRNA levels correlated with both *RORC* (p < 0.002 and p < 0.0001, respectively) and *FOXP3* (p < 0.0001 and p < 0.005) mRNA levels (Fig. 5b, d). On the other hand, *SOCS1* mRNA levels positively correlated with the mRNA expression of *CDKN2A (p14*^*Arf*^*), CDKN2B (p15*^*Ink4b*^*)* and *CDKN2BAS* (Fig. 6a, p < 0.03, p < 0.0001 and p < 0.0003, respectively). *SOCS3* mRNA levels correlated with *CDKN2B (p15*^*Ink4b*^) but not with the other gene mRNA expression levels (Fig. 6b p < 0.02).

## Discussion

T1DM patients have an increased risk of CVD but the causes of this risk are not fully understood. In the present study, we found diminished mRNA expression levels of *CDKN2A (p16*^*Ink4a*^*)*, *CDKN2A (p14*^*Arf*^), *CDKN2B* (*p15*^*Ink4b*^) and *CDKN2BAS* genes in circulating leukocytes of T1DM patients, who also displayed increased atherosclerosis risk measured as CC-IMT. Consistent with the well-known deranged immune T-cell function, T1DM subjects exhibited lower circulating Treg (CD4+CD25+CD127−) cell percentages and reduced mRNA levels of transcription factors related to CD4+ Th differentiation, *RORC and FOXP3,* which are determinants for Th17 and Treg differentiation, respectively. In agreement with lower expression of these transcription factors, IL2 and IL6 cytokine levels were also decreased in T1DM patients. Notably, T1DM subjects had enhanced percentages of the proinflammatory CD14++CD16+ monocyte subpopulation, a cellular subset that has been reported to predict acute cardiovascular events. Altogether, our study indicates an association between increased atherosclerosis in T1DM and reduced expression of *CDKN2A/2B/2BAS* in circulating leukocytes which displayed proatherogenic phenotypes such as enhanced proinflammatory monocytes and reduced Treg content. Therefore, the present data suggest for the first time a potential role of *CDKN2A/2B/2BAS* through a decreased expression in leukocytes in atherosclerosis risk and development in T1DM, which could facilitate a pro-atherogenic profile in these cells by generating proinflammatory monocytes and lower content of Treg cell content.

Former studies have shown an increased CVD risk in T1DM patients despite intensive glycemic control [4]. However, ours is the first investigation that associates this CVD risk with a markedly reduced expression of *CDKN2A/2B/2BAS* genes in circulating leukocytes from T1DM patients. Previous genome-wide studies have linked SNPs in *CDKN2A/2B/2BAS* genes with enhanced risk of CVD and with T2DM [11]. In addition, functional human and murine studies have shown a protective role of these genes against metabolic diseases. An atheroprotective function has therefore been suggested for these genes, since genetic inactivation of *Cdkn2a* variants in mice increases atherosclerosis [12, 15] and reduced expression of the transcripts has been found in atherosclerosis patients [16]. Other studies suggested a role of these genes in carbohydrate metabolism [24], in insulin secretion [14] and β-cell islet biology [25]. Enhanced expression of *Cdkn2a/2b* in mice also prevents insulin resistance associated with aging [13] and delays hepatic steatosis produced by insulin resistance [17]. In line with these investigations, our study indicates an association between diminished expression of *CDKN2A/2B/2BAS* genes in leukocytes and increased atherosclerosis risk in T1DM.

One limitation in our study is the higher frequency of statin use as lipid-lowering strategy which might exert anti-inflammatory actions [26]. However, the use of these did not interfere with the study as T1DM subjects displayed enhanced inflammatory status with increased proinflammatory monocytes and decreased Treg cells.

In our study enhanced CC-IMT was also accompanied by augmented BMI and glucose levels and decreased circulating HDL-C which is consistent with the well-known association between altered metabolism and CVD. These metabolic abnormalities could modify CVD risk in T1DM patients as seen in T2DM subjects. However, this risk could be further enhanced by the intrinsic dysregulation of T cells of T1DM subjects. T1DM is characterized by impaired Treg function [27] and reduced circulating Treg cells have been found in humans with coronary atherosclerosis, acute coronary syndromes and plaque rupture [8, 9]. Therefore, reduced circulating Treg cells in our T1DM subjects could reasonably contribute to increase atherosclerosis. On the other hand, deranged balance of CD4 + T cell subsets whose lineage commitment is specified by cytokine environment and activation of their corresponding transcription factors [28] might also promote atherosclerosis [9, 29, 30]. T1DM patients exhibited decreased levels of IL2 and IL6 cytokine, which in the presence of TGFβ, promote Treg differentiation by inducing *FOXP3* expression and Th17 differentiation through *RORC* expression, respectively. *FOXP3* and *RORC* were consistently diminished in leukocytes in our T1DM patients thus indicating reduced levels of Treg like before, as well as decreased Th17, whose role in atherosclerosis is controversial [30]. Moreover, *SOCS1*, which negatively regulates Treg but is necessary for its function [23] was also significantly reduced in T1DM subjects. Besides modulation of self-tolerance, Treg cells promote an anti-inflammatory macrophage phenotype and cytokines [6], hence defective function or number of these could contribute to generate proinflammatory monocytes/macrophages. In fact, a major finding of our study is that increased atherosclerosis in T1DM was associated with a prevalence of circulating proinflammatory CD14++CD16+ monocytes which have been shown to predict cardiovascular events independently of other risk factors [21] and are associated with coronary plaque vulnerability in coronary disease patients [31]. We did not observe changes in the pro-inflammatory CD14++ monocytes which is consistent with another a study showing that CD14++CD16− monocytes do not associate with atherosclerosis measured as CC-IMT in subjects without CV acute events [32]. To our knowledge, this is the first study to indicate a relationship between CD14++CD16+ monocyte prevalence and atherosclerosis in T1DM.

Pro-atherogenic and proinflammatory leukocyte phenotypes observed in T1DM subjects, although promoted by the autoimmune cell derangement characteristic of these subjects, could be aggravated by the diminished expression of *CDKN2A/2B/2BAS* genes. Supporting this, a positive correlation was observed between *CDKN2A/2B/2BAS* expression and the transcription factors *FOXP3*, *RORC* and *SOCS3* mRNA levels in leukocytes, suggesting a potential relationship between them. Furthermore, several lines of evidences from previous research support a role of these genes in immune cell modulation. Thus, an anti-inflammatory role has been attributed to *Cdkn1a* (variant 1, p16^Ink4a^) by promoting IRAK1 degradation and diminishing cytokine secretion in macrophages [33]. Increased expression of *Cdkn2a/2b* genes in mouse models decreased circulating proinflammatory Ly6C^hi^ monocytes, reduced activated T cells and prevented macrophage infiltration into tissues [17]. Genetic inactivation of *Cdkn2a* in the myeloid lineage enhanced Ly6C^hi^-monocytes [15], promoted megakaryopoiesis and increased platelet activity [34]. Local infusion of *Cdkn2a* (variant 1) in bone joints consistently impaired the expression of proinflammatory cytokines in a mouse model of autoimmune rheumatoid arthritis [35]. Notably, we recently reported that coronary artery disease in T2DM (T2DM-CAD subjects) is accompanied by decreased Treg cells and diminished expression of *CDKN2A/2B/2BAS* [10]. Furthermore, treatment of human lymphocytes in vitro and of atherosclerotic mouse models in vivo with PD0332991, a p16^Ink4a^/p15^Ink4b^ mimetic drug and a proven selective inhibitor of CDK4 (the main target of these proteins), augmented Treg levels and diminished atherosclerosis and vulnerable plaque in the mouse model [10]. Comparison of both investigations revealed a much lower *CDKN2A/2B/2BAS* gene expression in our former study in T2DM and T2DM with CAD compared with controls, which were up to 60-80% reductions while T1DM subjects displayed 40% reductions compared with controls. In addition, CV risk measured as CC-IMT were 0.46, 0.594 and 0.829 for T1DM, T2DM and T2DM-CAD respectively which correlates well with reduced expression of *CDKN2A/2B/2BAS* genes and is consistent with the protective role of these genes. Altogether, these findings suggest that *CDKN2A/2B/2BAS* gene modulation might modify inflammatory cell phenotype in diverse experimental settings. Hence, decreased expression of *CDKN2A/2B/2BAS* genes in the present study could potentially contribute to the altered leukocyte phenotype and promote atherosclerosis in T1DM subjects.

## Conclusions

Our study shows for the first time that increased atherosclerosis risk in T1DM patients associates with decreased expression of *CDKN2A/2B/2BAS* in leukocytes, which display pro-atherogenic phenotypes consisting of reduced circulating Treg cells and augmented proinflammatory CD14++ CD16+ monocytes. Expansion and stabilization of Tregs have been cited as promising therapies to treat both atherosclerosis [7] and autoimmune diseases such as T1DM [27]. Altogether, this suggests that therapies targeted at increasing *CDKN2A/2B/2BAS* to restore Treg cells, such as the ones we have previously described [10], could potentially be used to reduce CVD in T1DM patients.

## Additional file

**Additional file 1.** Tables for correlation studies: Table S1 and Table S2.

## Data Availability

The datasets used and analyzed during the current study cannot be public available due to the individual privacy of the subjects included in the study. However, the data generated and included in the current study are available from the corresponding author upon reasonable request.

## Abbreviations

BW: body weight
CAD: coronary artery disease
CDK: cyclin-dependent kinase
CVD: cardiovascular disease
Th: T helper cell
Treg: regulatory T cell
