# Supplementary material for: Type 1 diabetic mellitus patients with increased atherosclerosis risk display decreased CDKN2A/2B/2BAS gene expression in leukocytes
Source: J Transl Med. 2019 Jul 12;17:222. doi: 10.1186/s12967-019-1977-1 (PMC6626385; doi:10.1186/s12967-019-1977-1)
Supplement: Supplementary file 1 — Additional file 1. Tables for correlation studies: Table S1 and Table S2. [file 12967_2019_1977_MOESM1_ESM.docx]

**Additional File 1**

**Type 1 Diabetic mellitus patients with increased atherosclerosis display decreased *CDKN2A/2B/2BAS* gene expression in leukocytes**

Sergio Martínez-Hervás^a,b,c^, Verónica Sánchez-García^b^, Andrea Herrero-Cervera^b^, Ángela Vinué ^b^, José Tomás Real^a,b,c^, Juan F Ascaso^a,b,c^, Deborah Jane Burks^c,d^, Herminia González-Navarro^b,c,e,*^

^a^ Endocrinology and Nutrition Department Hospital Clínico Universitario. Department of Medicine, University of Valencia, 46010 Valencia, Spain

^b^ INCLIVA Institute of Health Research, 46010 Valencia, Spain

^c^ CIBER Diabetes and Associated Metabolic Diseases (CIBERDEM), 28029 Madrid, Spain

^d^ Príncipe Felipe Research Center (CIPF), 46012 Valencia, Spain

^e^ Department of Didactics of Experimental and Social Sciences. University of Valencia, 46010 Valencia, Spain

^*^**Correspondence**:

Herminia González-Navarro, INCLIVA Institute of Health Research

Avda. Menéndez Pelayo, 4, 46010, Valencia (Spain)

Phone: +34-96 3864403; Fax: +34-96 1973540

E-mail: [herminia.gonzalez@uv.es](mailto:herminia.gonzalez@uv.es)

**Keywords:** type 1 diabetes, inflammation, cardiovascular risk, T cells

**Additional Files**

**Table S1. Correlation studies of demographic and plasmatic parameters with CC-IMT.**

|  | **Rho Spearman** | | | **p value** | | | |
| --- | --- | --- | --- | --- | --- | --- | --- |
| **AGE** | | | 0.326 | | 0.025* | |  |
| **BMI** | | | 0.348 | | 0.030* | |  |
| **Glucose levels** | | | 0.349 | | 0.010* | |  |
| **HbA1C levels** | | 0.354 | | | | 0.011* |  |
| **TOTAL-C levels** | | 0.266 | | | | 0.054 |  |
| **LDL-C levels** | | 0.314 | | | | 0.022* |  |
| **HDL-C** | | -0.092 | | | | 0.512 |  |
| **apoB levels** | | 0.3461 | | | | 0.011* | |

BMI: body mass index; TOTAL-C: total cholesterol; LDL-C: low density lipoprotein cholesterol; HDL-C: high density lipoprotein cholesterol; apoB: apolipoprotein B; CC-IMT: common carotid intima-media thickness; *p<0.05; Statistical significance was assessed by non-parametric Spearman correlation coefficient.

**Table S2. Correlation between HbA1C and glucose levels and mRNA expression levels CD4+ differentiation transcription factors**

|  | | **HbA1C** | | | | | |  |
| --- | --- | --- | --- | --- | --- | --- | --- | --- |
|  | | | **Rho spearman** | **p value** | | | | |
| ***TBET*** | -0.1831 | | | | 0.1542 | |  |  |
| ***GATA3*** | -0.1344 | | | | 0.3019 | | | |
| ***RORC*** | -0.2433 | | | | 0.0589 | | | |
| ***FOXP3*** | -0.6124 | | | | 0.0061* | | | |
| ***SOCS1*** | -0.2477 | | | | 0.0503* |  |  |  |
| ***SOCS3*** | -0.0709 | | | | 0.5867 | | | |
|  | | **Glucose** | | | | | | |
|  | | | **Rho spearman** | **p value** | | | | |
| ***TBET*** | -0.0962 | | | | 0.4495 | |  |  |
| ***GATA3*** | -0.1709 | | | | 0.1840 | | | |
| ***RORC*** | -0.09889 | | | | 0.4407 | | | |
| ***FOXP3*** | -0.2803 | | | | 0.0486* | | | |
| ***SOCS1*** | -0.3549 | | | | 0.0037* |  |  |  |
| ***SOCS3*** | -0.02655 | | | | 0.8363 | | | |

*p<0.05; Statistical significance was assessed by non-parametric Spearman correlation coefficient.

|  |  |  |
| --- | --- | --- |
